# Supplementary material for: The Evolution and Characterization of the RNA Interference Pathways in Lophotrochozoa
Source: Genome Biol Evol. 2024 May 7;16(5):evae098. doi: 10.1093/gbe/evae098 (PMC11114477; doi:10.1093/gbe/evae098)
Supplement: evae098_Supplementary_Data [file evae098_supplementary_data.zip › Supplementary_materials.zip!/selected_miRNAs/NoGe_selected_miRNAs/mirdeep_pdf_files/NMRB01005527.1_Notospermus_geniculatus_strain_Ushimado_scaffold5527,_whole_genome_shotgun_sequence_47695.pdf]

Provisional ID	:	NMRB01005527.1_Notospermus_geniculatus_strain_Ushimado_scaffold5527,_whole_genome_shotgun_sequence_47695
Score total	:	119.1
Score for star read(s)	:	3.9
Score for read counts	:	113.3
Score for mfe	:	2.5
Score for randfold	:	0
Score for cons. seed	:	-0.6
Total read count	:	234
Mature read count	:	156
Loop read count	:	0
Star read count	:	78

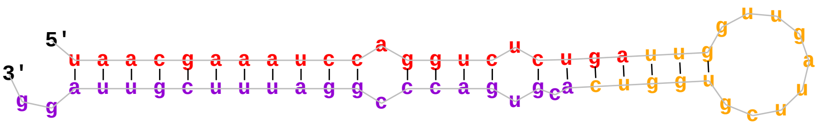

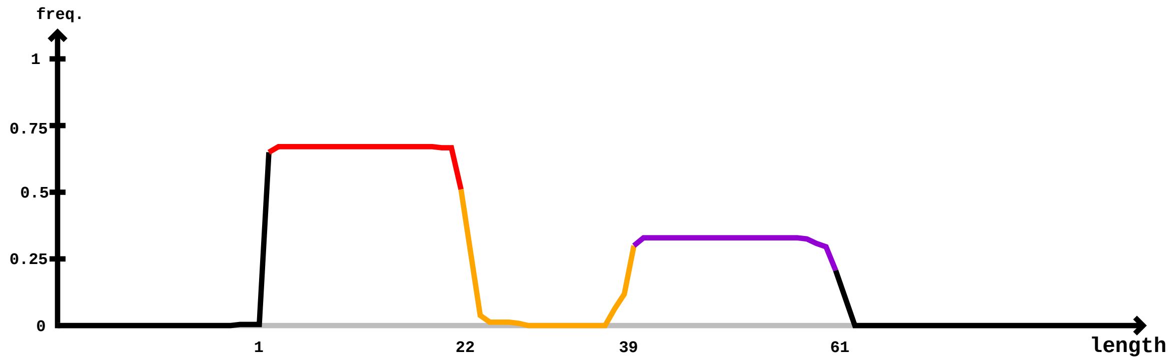

Mature Star

```
5'- gcgaugaccuguaccugucc uaacgaaauccaggucucugauugguugauucguggucacgugacccggauuucguuaggccagguacagguacgucgacaagauggcggc -3'   obs
```

```
    gcgaugaccuguaccuguccuaacgaaauccaggucucugauugguugauucguggucacgugacccggauuucguuaggccagguacagguacgucgacaagauggcggc        exp
    .((((((((((((((((.(((((((((((((.((((.(((((((.........)))))).).)))).))))))))))))).))))))))))).)))))............. reads  mm  sample
    .................uccuaacgaaaGccaggucucugauuggu.................................................................  1      1   Mm3
    ....................uaacgaaauccGggucucug.......................................................................  1      1   Mm3
    ....................uaacgaaauccaggucucug.......................................................................  24     0   Mm3
    ....................uaacgaaaucAaggucucuga......................................................................  1      1   Mm3
    ....................uaacgaaauccaggucucugG......................................................................  2      1   Mm3
    ....................uaacgaaGuccaggucucuga......................................................................  1      1   Mm3
    ....................uaacgaaauccaggucucuga......................................................................  36     0   Mm3
    ....................uaacgaaauccGggucucuga......................................................................  22     1   Mm3
    ....................uaacgaaauccaggucCcugau.....................................................................  1      1   Mm3
    ....................uaacgaaauccaggucucugaA.....................................................................  4      1   Mm3
    ....................uaacgaaauccaggucucugau.....................................................................  13     0   Mm3
    ....................uaacgaaauccGggucucugau.....................................................................  4      1   Mm3
    ....................uaacgaaauccaggucuUugau.....................................................................  1      1   Mm3
    ....................uaacgaaauccaggucAcugau.....................................................................  1      1   Mm3
    ....................uaacgaaGuccaggucucugau.....................................................................  1      1   Mm3
    ....................uaacgaaauccaggucucugaAu....................................................................  2      1   Mm3
    .....................aacgaaauccaggucucugauu....................................................................  1      0   Mm3
    ........................................................ucacgugacccggauuucguuag................................  2      0   Mm3
    ........................................................ucacgugacccggauuucguuagU...............................  1      1   Mm3
    .........................................................cacgugacccggauuucguuag................................  4      0   Mm3
    .........................................................cacgugacccggauuucAuuag................................  1      1   Mm3
    .........................................................cacgugacccggauuucguuagU...............................  2      1   Mm3
    ..........................................................acgugacccggauuucguuag................................  5      0   Mm3
    ..........................................................acgugacccggauuucgAuagg...............................  1      1   Mm3
    ..........................................................acgugacccggauuucguuagU...............................  5      1   Mm3
    ..........................................................acgugacccggauuucguuagg...............................  4      0   Mm3
    ..........................................................acgugacccggauuucguuagA...............................  3      1   Mm3
    ..........................................................acgugacccggauuucguuaggc..............................  7      0   Mm3
    ..........................................................acgugacccggauuucguuGggc..............................  3      1   Mm3
    ...........................................................cgugacccggauuucguuaggc..............................  4      0   Mm3

    ....................uGacgaaauccaggucucugau.....................................................................  5      1   Mm1
    ....................uaacgaaauccaggucucugau.....................................................................  2      0   Mm1
```

```
                    Mature                             Star
gcgaugaccuguaccuguccuaacgaaauccaggucucugauugguugauucgugguc acgugacccggauuucguuaggccagguacagguacgucgacaagauggcggc

....................uaacgaaauccaggucuc.........................................................................   1    0   Mm2
....................uaacgaaauccaggucucug.......................................................................   12   0   Mm2
....................uaacgaaGuccaggucucuga......................................................................   2    1   Mm2
....................uaacgaaauccaggucucuga......................................................................   5    0   Mm2
....................uaacgaaauccGggucucuga......................................................................   5    1   Mm2
....................uaacgaaauccaggucucugaA.....................................................................   2    1   Mm2
....................uaacgaaauccaggucucugau.....................................................................   1    0   Mm2
....................uaacgaaauccGggucucugau.....................................................................   1    1   Mm2
....................uaacgaaauccGggucucugauu....................................................................   1    1   Mm2
....................uaacgaaauccGggucucugauugguu................................................................   1    1   Mm2
....................uaacgaaaGccaggucucugauugguu................................................................   1    1   Mm2
.....................aacgaaauccGggucucugau.....................................................................   1    1   Mm2
.....................aacgaaauccaggucucugauu....................................................................   2    0   Mm2
........................................................ucacgugacccggauuucguua.................................   2    0   Mm2
........................................................ucacgugacccggauuucguuU.................................   1    1   Mm2
........................................................ucacgugacccggauuucguAag................................   1    1   Mm2
........................................................ucacgCgacccggauuucguuag................................   1    1   Mm2
.........................................................cacgugacccggauuucgu...................................   1    0   Mm2
.........................................................cacgCgacccggauuucguuag................................   1    1   Mm2
.........................................................cacgugacccggauuuUguuagg...............................   1    1   Mm2
.........................................................cacgugacccggauuucguuagA...............................   1    1   Mm2
..........................................................acgugacccggauuucguuag................................   1    0   Mm2
..........................................................acgCgacccggauuucguuag................................   2    1   Mm2
..........................................................acgugacccggauuucguuagg...............................   5    0   Mm2
..........................................................acgCgacccggauuucguuagg...............................   6    1   Mm2
..........................................................acgugacccggauuucguuaggc..............................   1    0   Mm2
...........................................................cgugacccggauuucguuaggc..............................   3    0   Mm2

.....................aacgaaauccaggucucuga......................................................................   1    0   Fm3
........................................................ucacgugacccggauuucguu..................................   4    0   Fm3
........................................................ucacgugacccggauuucguuaU................................   3    1   Fm3
.........................................................cacgugacccggauuucguuagU...............................   2    1   Fm3
```
